# Plasma biomarkers of small intestine adaptations in obesity-related metabolic alterations

**DOI:** 10.1186/s13098-020-00530-6

**Published:** 2020-04-09

**Authors:** Catherine Lalande, Jean-Philippe Drouin-Chartier, André J. Tremblay, Patrick Couture, Alain Veilleux

**Affiliations:** 1 École de nutrition, Faculté des sciences de l’agriculture et de l’alimentation, Université Laval, 2440, boulevard Hochelaga, Québec, QC G1V 0A6 Canada; 2 Centre Nutrition, santé et société (NUTRISS), Institut sur la nutrition et les aliments fonctionnels (INAF), Université Laval, Québec, QC Canada; 3 Centre des maladies lipidiques, Centre Hospitalier Universitaire (CHU) de Québec, Québec, QC Canada; 4 Centre de Recherche de l’Institut Universitaire de Cardiologie et de Pneumologie de Québec, Québec, QC Canada; 5 Canada Excellence Research Chair in the Microbiome-Endocannabinoidome Axis in Metabolic Health, Québec, QC Canada

**Keywords:** Citrulline, I-FABP, Small intestine, Insulin resistance, Type 2 diabetes, Adiposity

## Abstract

**Background:**

Evidence suggests that pathophysiological conditions such as obesity and type 2 diabetes (T2D) are associated with morphologic and metabolic alterations in the small intestinal mucosa. Exploring these alterations generally requires invasive methods, limiting data acquisition to subjects with enteropathies or undergoing bariatric surgery. We aimed to evaluate small intestine epithelial cell homeostasis in a cohort of men covering a wide range of adiposity and glucose homoeostasis statuses.

**Methods:**

Plasma levels of citrulline, a biomarker of enterocyte mass, and I-FABP, a biomarker of enterocyte death, were measured by UHPLC‑MS and ELISA in 154 nondiabetic men and 67 men with a T2D diagnosis.

**Results:**

Plasma citrulline was significantly reduced in men with insulin resistance and T2D compared to insulin sensitive men. Decreased citrulline levels were, however, not observed in men with uncontrolled metabolic parameters during T2D. Plasma I-FABP was significantly higher in men with T2D, especially in presence of uncontrolled glycemic and lipid profile parameters. Integration of both parameters, which estimate enterocyte turnover, was associated with glucose homeostasis as well as with T2D diagnosis. Differences in biomarkers levels were independent of age and BMI and glucose filtration rates.

**Conclusions:**

Our study supports a decreased functional enterocyte mass and an increased enterocyte death rate in presence of metabolic alterations but emphasizes that epithelial cell homeostasis is especially altered in presence of severe insulin resistance and T2D. The marked changes in small intestine cellularity observed in obesity and diabetes are thus suggested to be part of gut dysfunctions, mainly at an advanced stage of the disease.

## Background

The small intestine is capable of important morphological and metabolic adaptations to respond to rapid changes in nutrient intake, stress or partial resection [1–3]. Recent evidence underlines inadequate intestinal adaptations in pathophysiological conditions such as obesity and type 2 diabetes (T2D). Obesity is associated with an increased small intestine mucosa surface (i.e., enterocyte mass, crypt depth and villi length) as well as an increased immune cell population [4, 5]. In presence of obesity and type 2 diabetes, small intestine metabolic functions also present several alterations such as increased epithelial permeability, oxidative stress damage and exaggerated secretion of triglyceride-rich lipoproteins [6–8]. Difficulties to obtain fresh small intestine samples from subjects devoid of enteropathies or severe obesity complicate the elucidation of the effect of weight, glucose homeostasis and lipid profile on small intestine morphology and metabolic functions. Thus, previous literature on small intestine morphologic and metabolic adaptations in obesity-related conditions is only considering severely obese population. Development of biomarkers may therefore represent an interesting strategy to assess these small intestine parameters in individuals with a large spectrum of adiposity and glucose homeostasis.

Citrulline is a key intermediate in the urea cycle but liver remains a negligible contributor to its circulating levels. Enterocytes are the main site of citrulline biosynthesis from dietary and plasma amino acid precursors (i.e., arginine, ornithine, glutamine, glutamate or proline) [9–13]. Citrulline catabolism remains stable in absence of kidney disease and the small intestine therefore appears as the most important determinant of plasma citrulline levels. Reduced plasma citrulline levels were also documented in conditions characterized with lower enterocyte mass such as celiac disease, acquired immunodeficiency syndrome (AIDS), small intestine transplantation and sepsis-induced intestinal dysfunctions [14–19].

Fatty acid binding proteins (FABP) are a family of proteins facilitating intracellular fatty acid transport. Expression of the intestinal-specific FABP (I-FABP) is strictly limited to the small intestine mucosa. Significant amount of I-FABP is observed in circulation and this phenomenon is attributed to the release of enterocyte cytosolic content upon apoptosis. Indeed, small intestine mucosa presents an elevated cellular turnover regenerating the epithelium every 3 to 5 days. Higher I-FABP circulating levels are observed in conditions associated with a higher enterocyte death rate such as intestinal ischemia, hematopoietic stem-cell transplant, necrotizing enterocolitis and sepsis-induced intestinal dysfunctions [16, 20–22].

Attempts to assess small intestine mucosa adaptations in the whole spectrum of adiposity and glucose homeostasis remain incomplete in human as only samples from subjects with severe obesity and late-stage T2D have been studied. Citrulline and I-FABP are interesting non-invasive biomarkers to explore small intestine mucosa morphology and local cellular turnover in the context of obesity and metabolic diseases. The aim of the study is to evaluate small intestine epithelial cell homeostasis in a cohort of men covering a wide range of adiposity and glucose homoeostasis statuses. We hypothesized that circulating citrulline and I-FABP levels can highlight small intestine mucosa morphologic alterations associated with adiposity and metabolic diseases.

## Methods

### Study population

The study cohort included 221 men covering a wide range of adiposity and glucose homeostasis values. Participants were recruited through the Institute of Nutrition and Functional Food (INAF) clinical unit (Laval University) in the context of several research projects [23–34]. Exclusion criteria included smoking, cardiovascular disease, cancer history, monogenic lipid disorder, altered endocrine or hepatic function, gastrointestinal disease, HIV infection, alcoholism and severe dyslipidemia. The study cohort included 154 nondiabetic men and 67 men previously diagnosed with T2D. To be part of the study, subject with T2D had to have received stable doses of metformin for at least 3 months before randomization of the clinical trials. Also, metformin had to be the only antidiabetic drug for at least 6 weeks before the beginning of the study. Subjects with diabetes had to be withdrawn from lipid-lowering medications during the run-in period of the clinical trials, the moment at which the samples were collected for this study. These projects were approved by the ethics committee of Laval University Medical Research Center.

### Anthropometric, lipid profile and glucose homeostasis

Baseline anthropometric data and overnight fasting blood were obtained during screening visits at INAF’s clinical investigation unit. Plasma was collected in EDTA tubes after centrifugation (3000 RPM, 10 min) and stored at − 80 °C. Glucose was assessed using the glucose oxidase method and insulin was quantified by electrochemiluminescence (Roche Diagnostics). The HOMA-IR index was calculated using the following formula: fasting insulin (µU/mL) × fasting glucose (mmol/L) ÷ 22.5 [35]. Serum cholesterol and triglyceride concentrations were determined with a Roche/Hitachi modular analyzer (Roche Diagnostics). The glomerular filtration rate (GFR) was calculated with the Chronic Kidney Disease Epidemiology Collaboration equation: 141 × min (serum creatinine (mg/dL))^−0.411^ × max (serum creatinine (mg/dL))^−1.209^ × 0.993^Age^ [36].

### I-FABP

Plasma I-FABP levels were measured in duplicate using the Human I-FABP DuoSet ELISA kit (R&D Systems, Minneapolis, MN) with some modifications to the manufacturer recommendation. Samples were diluted 1/20 in assay diluent (5 mM Tris, 150 mM NaCl,2.7 mM KCl, 10 mM Na^2^HPO^4^, 1.8 mM KH^2^PO^4^, 0.2 mM EDTA, 0.01% SDS, 0.05% sodium deoxycholate, 0.1% Triton X-100 and 2% BSA). Intra- and inter-assay coefficients were 6.6% (n = 223) and 10.6% (n = 20), respectively. The limits of detection (LOD) and limit of quantification (LOQ) were respectively of 80 and 300 pg/mL.

### Plasma amino acid profile

Plasma amino acids were extracted and derivatized using the EZ: FAAST kit (Phenomenex, USA) as specified by the manufacturer. Along with other internal standards, deuterated citrulline (100 μM) was included in the assay to optimize citrulline quantification in duplicate. Amino acid profiles were achieved using UHPLC coupled to Tandem Quadrupole Mass spectrometry (Acquity H-Class, Xevo TQD, Waters corp., Mildford, MA). Intra- and inter-assay coefficients were lower than 15% for all amino acids and were respectively of 2.6% (n = 244) and 3.7% (n = 18) for citrulline. Citrulline levels were above the LOQ (0.1 µmol/L) for all samples.

### Intestinal biopsies and gene expression

In a subset of nondiabetic men (n = 101), biopsies were collected from the second portion of the duodenum during gastroduodenoscopy in the fasting state [37]. Samples (3 × 3 mm) were collected using single-use biopsy forceps, immediately flash-frozen in liquid nitrogen, and stored at − 80 °C before RNA extraction. Intestinal tissue samples were homogenized in 1 mL of Qiazol (Qiagen, Hilden, Germany). RNA was extracted using a RNeasy kit (Qiagen). To eliminate any contaminating DNA, biopsies were treated with an RNase-free DNase set. Primer sequences for FABP2 were 5′‑TCAGGCTGGAATGTAGTGGAGAGA-3′ and 5′-CAAAACAAAAATTAGCTGGGCACTG-3′. Among housekeeping genes available in the data set, GDP6 was selected as it was not associated with any parameters analyzed [37].

### Statistical analyses

Differences in parameters between groups were tested using t-test and one-way ANOVA. Pearson’s correlation coefficients were computed to test plasma biomarkers associations with anthropometric and biochemical parameters. Potential confounding variables were assessed using generalized linear models. Non-normally distributed variables (Shapiro–Wilk p ≤ 0.05) were log_10_ or Box-Cox transformed. Differences between groups and associations were considered statistically significant at *p *< 0.05. Statistical analyses were performed using JMP Pro 12 software (SAS Institute, Cary, NC, USA).

## Results

Plasma I-FABP and citrulline levels were measured in a study sample of 221 lean to moderately obese men. Anthropometric and metabolic parameters of the study cohort are shown in Table 1. Men without diabetes (n = 154) were 37.6 ± 11.7 years old on average and were overweight according to a mean BMI of 29.8 ± 5.2 kg/m^2^. However, the sample was covering a wide range of BMI (20.0 to 43.1 kg/m^2^) and glucose homeostasis (HOMA-IR index: 0.8 to 10.0) values. Among these nondiabetics, we have identified 106 men with insulin resistance (IR), as defined by an HOMA-IR index greater than 2.5. These men with IR were mostly obese (31.5 ± 4.6 kg/m^2^) and characterized by an unfavorable glucose homeostasis and lipid profile (p < 0.05) as compared to men with relatively high insulin sensitivity (IS) (HOMA-IR index < 2.5; n = 48; Table 1). Men with T2D were older (56.8 ± 6.3 years) and mostly obese (BMI: 31.2 ± 4.9 kg/m^2^) on average. Compared to nondiabetic men, they were also characterized by an elevated fasting glycemia (8.5 ± 2.1 mmol/L) and a deteriorated plasma lipid profile.

**Table 1:** Physical and metabolic characteristics of the study sample (n = 221 men)

Variables	IS (n = 48) (mean ± SD)	IR (n = 106) (mean ± SD)	T2D (n = 67) (mean ± SD)
***Anthropometrics***			
Age (years)	35.3 ± 12.5	38.7 ± 11.2	56.8 ± 6.3^a,b^
BMI (kg/m^2^)	25.9 ± 4.5	31.5 ± 4.6^a^	31.2 ± 4.9^a^
Waist circumference (cm)	93 ± 14	109 ± 13^a^	106 ± 14^a^
***Glucose homeostasis***			
Fasting glucose (mmol/L)	5.0 ± 0.5	5.3 ± 0.5^a^	8.5 ± 2.1^a,b^
HbA1c (%)	–	–	7.0 ± 1.2
HOMA-IR	1.9 ± 0.4	4.5 ± 1.4^a^	–
Insulin (pmol/L)	58 ± 12	133 ± 39^a^	119 ± 69^a,b^
***Lipid profile***			
HDL cholesterol (mmol/L)	1.2 ± 0.2	1.0 ± 0.2^a^	1.0 ± 0.2^a^
LDL cholesterol (mmol/L)	3.1 ± 0.7	3.3 ± 0.9	3.6 ± 1.1^a^
TG (mmol/L)	1.3 ± 0.7	2.2 ± 1.0^a^	2.7 ± 1.5^a,b^
hsCRP (mg/L)	2.1 ± 2.2	4.0 ± 3.9	3.5 ± 4.0
Glomerular filtration rate (mL/min/m^2^)	104 ± 15	100 ± 13	94 ± 10^a,b^

^a^p < 0.05 vs IS; ^b^p < 0.05 vs IR

### Plasma amino acid profile

Total amount of plasma amino acids remains unaltered in men across the range of adiposity and glucose homeostasis status (Table 2). Plasma levels of branched amino acids were significantly increased in men with IR (466 ± 92 µmol/L) and T2D (479 ± 129 µmol/L) compared to men with IS (430 ± 76 µmol/L; p < 0.05) as previously reported [38, 39]. Plasma levels of branched amino acids were positively associated with BMI, waist circumference, HOMA-IR, TG and C-reactive protein (0.19 < r < 0.25; n = 153; p < 0.05) in subjects without diabetes. Similarly, aromatic amino acid levels were higher in men with IR and T2D compared to men with IS and positively associated with BMI, HOMA-IR and TG (0.22 < r < 0.30; n = 153; p < 0.05) in these subjects, which is also concordant with previous study. The complete plasma amino acid profile in each subgroup as well as individual correlation with HOMA-IR is included in Table 2.

**Table 2:** Plasma amino acid profile in men (n = 221)

Amino acids (μmol/L)	IS (n = 48) (mean ± SD)	IR (n = 106) (mean ± SD)	T2D (n = 67) (mean ± SD)	HOMA-IR^#^ (r values)
***Individual amino acids***				
Alanine (Ala)	291 ± 73	318 ± 81	338 ± 107^a^	0.22*
Arginine (Arg)	55.0 ± 14.4	55.6 ± 12.2	49.7 ± 12.9^a,b^	0.08
Asparagine (Asn)	39.9 ± 6.6	37.2 ± 6.4^a^	38.1 ± 7.4	− 0.21*
Aspartic acid (Asp)	5.42 ± 2.52	5.42 ± 1.99	5.71 ± 1.88	0.10
*Citrulline (Cit)*	26.5 ± 4.6	24.1 ± 5.0^a^	23.3 ± 6.7^a^	− 0.21*
Cysteine (Cys)	4.26 ± 2.36	4.77 ± 2.18	4.80 ± 2.19	0.19*
Glutamine (Gln)	526 ± 104	509 ± 80	469 ± 81^a,b^	− 0.06
Glutamic acid (Glu)	64.8 ± 36.4	83.1 ± 36.6^a^	84.6 ± 21.4^a^	0.32**
Glycine (Gly)	213 ± 43	184 ± 41^a^	179 ± 58^a^	− 0.26*
Histidine (His)	91.0 ± 14.2	89.5 ± 14.6	83.4 ± 16.7^a,b^	− 0.03
Isoleucine (Ile)	65.6 ± 16.8	70.4 ± 15.2	75.2 ± 21.8^a^	0.25*
Leucine (Leu)	123 ± 24	135 ± 24^a^	145 ± 39^a^	0.29**
Lysine (Lys)	149 ± 28	152 ± 29	155 ± 34	0.12
Methionine (Met)	21.1 ± 2.6	21.6 ± 2.9	20.8 ± 3.8	0.16*
Ornithine (Orn)	47.4 ± 12.7	45.3 ± 11.7	47.0 ± 15.5	− 0.04
Phenylalanine (Phe)	42.9 ± 7.4	46.5 ± 7.3^a^	47.5 ± 8.3^a^	0.24*
Proline (Pro)	186 ± 48	204 ± 52^a^	205 ± 64	0.18*
Serine (Ser)	76.4 ± 15.7	70.6 ± 12.1^a^	73.9 ± 15.1	− 0.07
Threonine (Thr)	99.8 ± 18.3	92.1 ± 16.6^a^	94.0 ± 23.4	− 0.13
Tryptophane (Trp)	56.9 ± 14.8	58.0 ± 13.2	55.8 ± 12.0	0.11
Tyrosine (Tyr)	49.5 ± 10.0	57.0 ± 10.5^a^	56.4 ± 13.1^a^	0.43**
Valine (Val)	241 ± 46	261 ± 59^a^	259 ± 75	0.21*
***Amino acid class***				
Aromatic	240 ± 32	251 ± 27^a^	243 ± 36	0.27**
Branched-chain	430 ± 76	466 ± 92^a^	479 ± 129^a^	0.25*
Citrulline precursors	879 ± 134	897 ± 109	856 ± 133^b^	0.11
Total amino acids	2517 ± 251	2530 ± 296	2518 ± 411	0.09
***Amino acid ratios***				
Citrulline/amino acids	0.011 ± 0.002	0.010 ± 0.002^a^	0.009 ± 0.003^a^	− 0.22*
Citrulline/precursors	0.031 ± 0.009	0.027 ± 0.006^a^	0.028 ± 0.008^a^	0.25*
Arg bioavailability	0.77 ± 0.24	0.83 ± 0.21	0.74 ± 0.21^b^	0.11
NO activity	0.51 ± 0.15	0.45 ± 0.12^a^	0.49 ± 0.21	0.13
Arginase activity	0.96 ± 0.49	0.86 ± 0.34	1.02 ± 0.58^b^	− 0.05
Arginase/NO activity	1.83 ± 0.56	1.97 ± 0.78	2.14 ± 0.88^a^	0.13

Citrulline precursors: Arg, Orn, Gln, Glu and Pro; Arg bioavailability: Arg/(Orn + Cit); NO activity: Cit/Arg; arginase activity: Orn/Arg; arginase/NO activity: Orn/Cit

^a^p < 0.05 vs IS; ^b^p < 0.05 vs IR ^#^Pearson correlation coefficient adjusted for age and GFR; *p < 0.05; **p < 0.001

### Citrulline

**Fig. 1:**
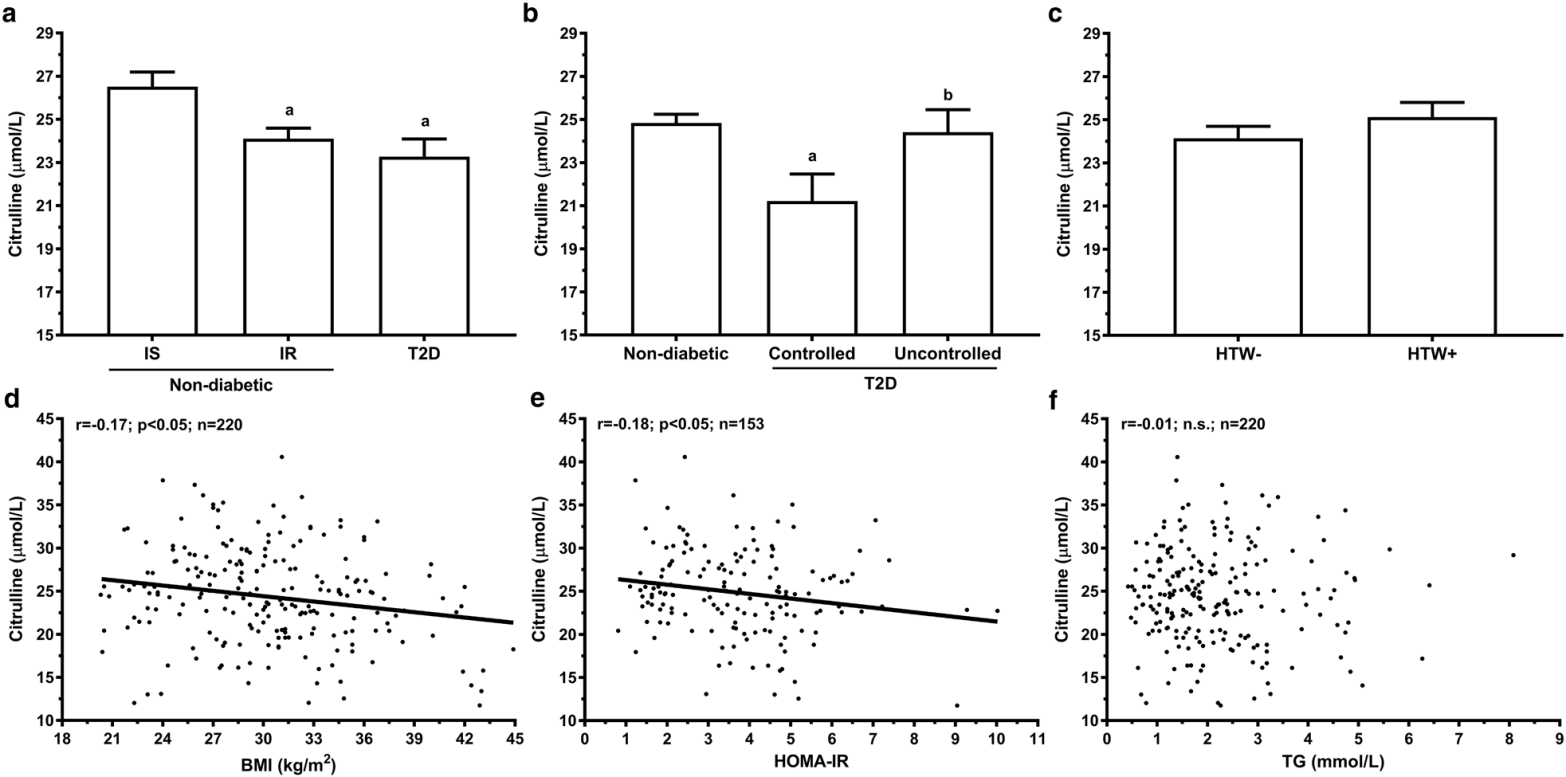
Plasma citrulline levels associations with anthropometric and metabolic parameters in men. Mean plasma citrulline levels (± SEM) according to **a** glucose homeostasis status, **b** T2D diagnosis and metabolic control (HbA1c < 6% and TG < 1.7 mmol/L) and **c** hypertriglyceridemic waist phenotype (HTW). Plasma citrulline levels correlation between **d** BMI, **e** HOMA-IR in subjects without T2D and **f** TG levels. Pearson correlation coefficient and p values of log_10_ or Box-Cox transformed variables are indicated are shown. ^a^p < 0.05 vs IS, nondiabetic or HTW; ^b^ p < 0.05 vs IR

Considering that citrulline biosynthesis is relying on the enterocyte mass, we have further analyzed plasma levels of this amino acid in these subjects. Plasma citrulline levels of men with IR (24.1 ± 5.0 µmol/L) were lower than those of men with IS (26.5 ± 4.6 µmol/L; p < 0.01), but similar to those of men with a T2D diagnosis (23.3 ± 6.7 µmol/L; Fig. 1a). Within the T2D subgroup, plasma citrulline levels were lower in subjects presenting controlled HbA1c (< 6%) and TG (< 1.7 mmol/L) levels compared to subject with abnormal values in either one or both parameters (Fig. 1b). Stratification according to the hypertriglyceridemic waist phenotype (HTW: Waist circumference > 90 cm and TG > 1.7 mmol/L) reveals higher plasma citrulline levels in men with T2D and HTW, but this difference was not observed in men without diabetes.

Plasma citrulline levels were negatively correlated with BMI (r = − 0.17; p < 0.05; n = 220), and GFR (r = − 0.25; p < 0.001; n = 215) in the whole sample of men (Fig. 1d, f). In nondiabetics, plasma citrulline levels were negatively associated with BMI, HOMA-IR index, CRP (− 0.20 > r > -0.17; p < 0.05; n = 118) and GFR (r = − 0.32; p < 0.001; n = 148; Fig. 1e). These associations remained significant in men without diabetes following adjustment for age and GFR, suggesting that the reduced plasma citrulline levels reflect a reduced small intestinal production rather than change in renal function. After further adjustment for BMI, plasma citrulline lost its association with HOMA-IR, but not with CRP (data not shown). In contrast, plasma citrulline levels in men with T2D were not associated with anthropometric and metabolic parameters except for a negative correlation with GFR (r = − 0.27; p < 0.01; n = 67). We performed linear regression models to identify independent predictor of plasma citrulline levels in men (Table 3). In the whole sample, GFR, BMI and T2D, explained 14.3% of the variance in plasma citrulline levels. When only men without diabetes were included in the analysis, GFR and HOMA‑IR explained more than 14% of citrulline level variance.

**Table 3:** Linear regression analyses predicting plasma biomarkers in men

Dependent variables	Independent variables	Partial^a^ (*r*^2^ × 100)	Total (*r*^2^ × 100)	*p* values
***Complete sample***				
Citrulline (µmol/L) (n = 214)	GFR (mL/min/m^2^)	8.9	14.3	p < 0.001
	BMI (kg/m^2^)	3.7		p < 0.01
	T2D diagnosis	1.7		p < 0.05
I-FABP (pg/mL) (n = 220)	T2D diagnosis	5.5	5.5	p < 0.001
I-FABP/citrulline (n = 218)	T2D diagnosis	9.9	9.9	p < 0.001
***Men without diabetes***				
Citrulline (µmol/L) (n = 147)	GFR (mL/min/m^2^)	10.5	14.5	p < 0.001
	HOMA-IR	4.0		p < 0.01
I-FABP (pg/mL) (n = 153)	–	–	–	NS
I-FABP/citrulline (n = 151)	HOMA-IR	3.2	3.2	p < 0.05

^a^Regression models included age, BMI, fasting glucose, GFR, HDL, LDL, T2D diagnostic and TG as well as HOMA-IR for analysis omitting men with T2D

We then explored the possibility that the associations of plasma citrulline levels with adiposity and metabolic parameters could be mediated by overall changes in total plasma amino acids and citrulline precursors (i.e., arginine, ornithine, glutamine, glutamate and proline). Citrulline-to-precursor amino acids and citrulline-to-total amino acids ratios were lower in the plasma of men with IR and T2D than of men with IS (p < 0.05; Table 2). These ratios were also correlated with BMI and HOMA-IR (− 0.26 < r < − 0.18; n = 153; p < 0.01; data not shown) in men without diabetes following adjustment for GFR and age, which strengthen the independent associations observed between plasma citrulline, adiposity and glucose homeostasis.

**Fig. 2:**
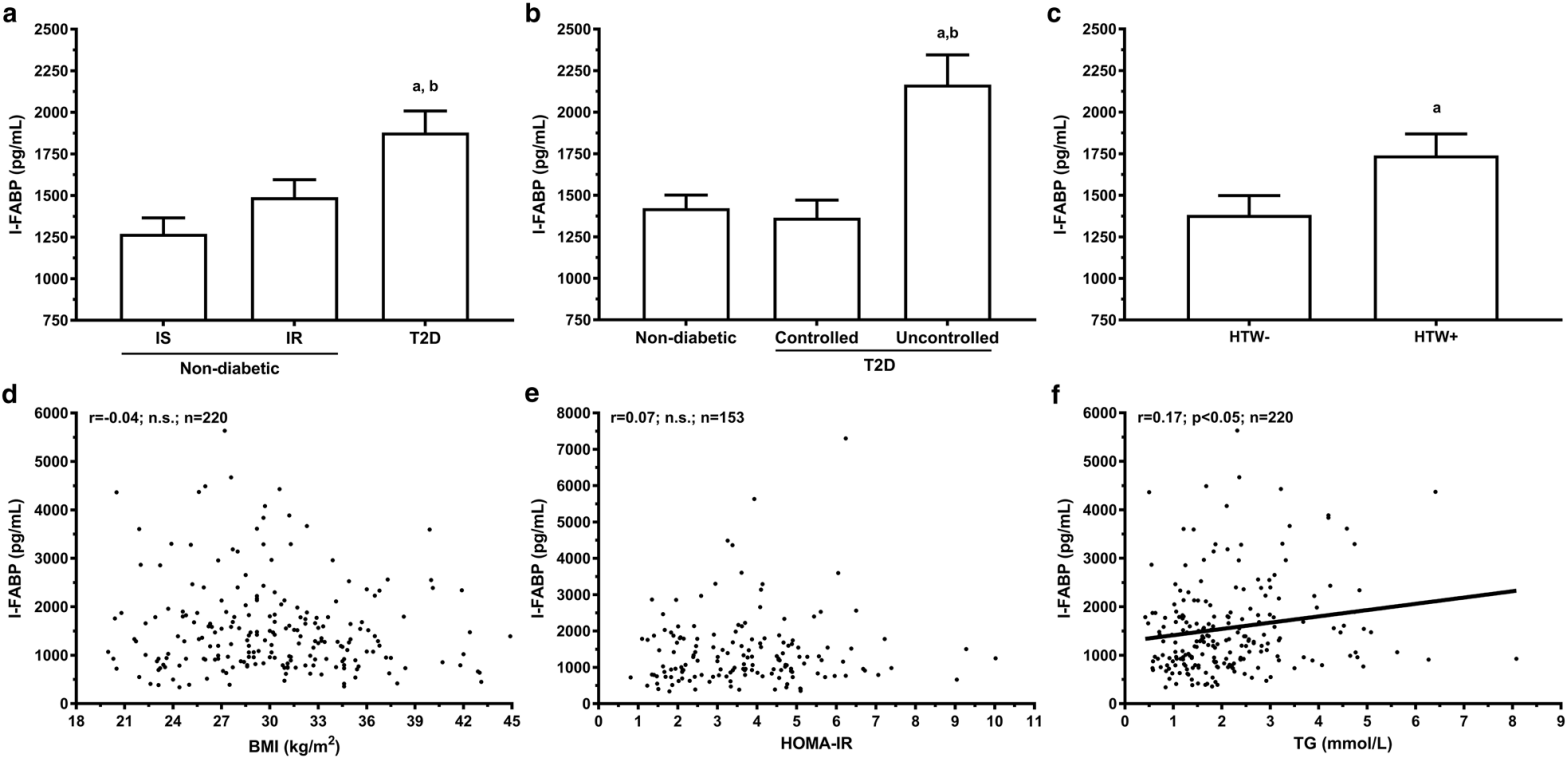
Plasma I-FABP levels associations with anthropometric and metabolic parameters in men. Mean I-FABP citrulline levels (± SEM) according to **a** glucose homeostasis status, **b** T2D diagnosis and metabolic control (HbA1c < 6% and TG < 1.7 mmol/L) and **c** hypertriglyceridemic waist phenotype (HTW). Plasma I-FABP levels correlation between **d** BMI, **e** HOMA-IR in subjects without T2D and **f** TG levels. Pearson correlation coefficient and p values of log_10_ or Box-Cox transformed variables are indicated are shown. ^a^p < 0.05 vs IS, nondiabetic or HTW; ^b^p < 0.05 vs IR

As described in the Table 2, citrulline precursors were either increased (glutamate and proline) or remained unaltered (arginine, glutamine and ornithine) in presence of insulin resistance in nondiabetics (p < 0.05). We also observed lower plasma arginine and glutamine levels in men with T2D. In sum, arginine bioavailability ratio (arginine/ornithine + citrulline) was slightly reduced in men with T2D while arginase activity and arginase-to-NO activity ratio were increased in men with T2D (Table 2). Nevertheless, citrulline levels remains independently associated with glucose homeostasis when adjusted for these ratios. These results confirm previous studies and suggest that change in citrulline levels are not due to important changes in arginine bioavailability [38] (Fig. 2).

### Plasma I-FABP

**Fig. 3:**
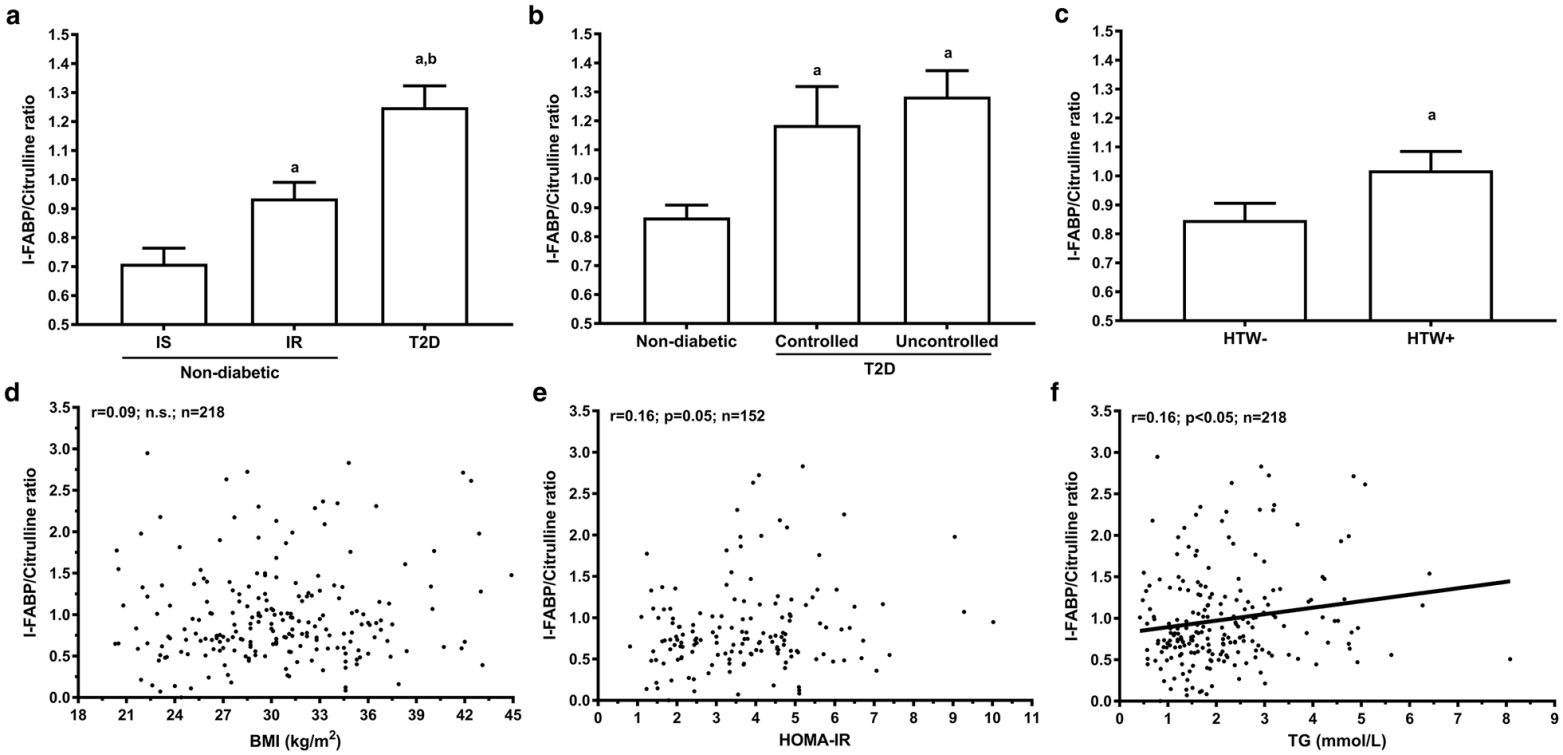
Plasma I-FABP-to-citrulline ratio associations with anthropometric and metabolic parameters in men. Mean plasma I-FABP-to-citrulline ratio (± SEM) according to **a** glucose homeostasis status, **b** T2D diagnosis and metabolic control (HbA1c < 6% and TG < 1.7 mmol/L) and **c** hypertriglyceridemic waist phenotype (HTW). Plasma I-FABP-to-citrulline ratio correlation between **d** BMI, **e** HOMA-IR in subjects without T2D and **f** TG levels. Pearson correlation coefficient and p values of log_10_ or Box-Cox transformed variables are indicated are shown. ^a^p < 0.05 vs IS, nondiabetic or HTW; ^b^p < 0.05 vs IR

Mean plasma I-FABP levels were significantly higher in men T2D (1879 ± 1056 pg/mL, p < 0.05) compared to men with IS (1269 ± 655 pg/mL) and IR (1434 ± 920 pg/mL; Fig. 2a). Interestingly, men with T2D with normal HbA1c (< 6%) or TG (< 1.7 mmol/L) showed similar plasma I-FABP levels than nondiabetics, while only those harboring an uncontrolled T2D had higher plasma I-FABP levels (Fig. 3b). The presence of insulin resistance, by itself, had no significant impact on plasma I-FABP levels in men without diabetes (Fig. 2a). In contrast, the hypertriglyceridemic waist phenotype was associated with significantly elevated levels of plasma I-FABP (Fig. 2c).

In the complete sample, plasma I-FABP levels correlated with CRP (r = − 0.17; p < 0.05; n = 184), fasting glucose (r = 0.25; p < 0.05; n = 220), and TG (r = 0.17; p < 0.05; n = 220), but not with BMI (Fig. 3d). As expected, these associations remained after adjustments for age and BMI. In men with T2D, plasma I-FABP correlated with CRP (r = − 0.34; p < 0.01; n = 67), and TG (r = 0.25; p < 0.05; n = 67; data not shown). In men without diabetes, plasma I-FABP showed no association with anthropometric and metabolic parameters. Linear regression models predicting plasma I-FABP levels in the complete sample of men reveal that T2D diagnosis explained 5.5% of the variance in plasma I-FABP (Table 3). In men without diabetes, there was no significant predictor of plasma I-FABP identified.

Duodenal FABP2 gene expression levels, available in a subset of the subjects without diabetes (n = 101), was not influenced by food intake, anthropometric or metabolic parameters (Additional file 1: Table S1). Plasma I-FABP levels correlate with duodenal FABP2 gene expression (r = 0.22; p < 0.05; n = 100) and this association was independent of adiposity and metabolic parameters. Adjustment of previous linear regression models with FABP2 gene expression lead to novel associations between circulating levels of I-FABP, BMI and HOMA-IR (p < 0.05). It suggests that changes in the plasma I-FABP pool are at least partly determined by duodenal expression levels, but its release tends to increase with BMI and glucose homeostasis alteration.

### I-FABP-to-citrulline ratio

Plasma I-FABP-to-citrulline ratio was calculated to integrate both cell renewal and enterocyte cell mass biomarkers. Mean plasma I-FABP-to-citrulline ratio was higher in men with T2D than in men with IR and IS (Fig. 3a). In men with IR, this ratio was also increased compared to men with IS, but remained significantly lower than men with T2D. The lipid profile and glycemic status in men with T2D have no impact on the plasma I‑FABP-to-citrulline ratio (Fig. 3b). In contrast, the presence of an hypertriglyceridemic waist phenotype was associated with increased plasma I-FABP-to-citrulline ratio in men without diabetes (Fig. 3c).

In the complete sample, plasma I-FABP-to-citrulline ratio was significantly correlated with fasting glucose and TG (0.16 > r = 0.28; p < 0.05; n = 218; Fig. 3d, f). Correlations with fasting glucose and TG remained significant after adjustment for age, BMI and GFR. In men without diabetes, plasma I-FABP-to-citrulline ratio correlate with HOMA-IR (r = 0.16; p < 0.05; n = 152; Fig. 3e). Following adjustment for age and GFR, HOMA-IR remain significantly associated with plasma I-FABP-to-citrulline ratio (r = 0.18, p < 0.05, n = 152), but was not independent of BMI. Finally, linear regression models shown that T2D diagnosis was the only independent contributor to explain the variance in plasma I-FABP-to-citrulline ratio. In men without diabetes, HOMA-IR explained only 4.8% of the variance in plasma I-FABP-to-citrulline ratio.

## Discussion

The portrait of small intestine mucosa adaptations in obesity and diabetes remains incomplete in human as only samples from subjects with severe disease states have been thoroughly characterized. Some studies have suggested the use of plasma citrulline and I-FABP levels as potential non-invasive biomarkers to explore small intestine mucosa morphology in enteropathies. We have investigated the associations of small intestine mucosa biomarkers with adiposity and metabolic parameters in a larger and well-characterized cohort of men (n = 221) covering a large spectrum of disease states. We found that plasma citrulline levels, a proxy of enterocyte mass, was negatively associated with adiposity, insulin resistance and diabetes but remain high in subjects with uncontrolled diabetes. Inversely, circulating levels of I-FABP, a proxy of enterocyte death rate, were higher in presence of insulin resistance and diabetes, particularly in subjects with inadequate glycemic and lipid control. These results suggest an increased enterocyte death rate with a lower absolute number of enterocytes in presence of insulin resistance. The increased enterocyte death rate was accompanied with a higher number of enterocytes in uncontrolled diabetic states, suggesting sustained cellular proliferation. Results also underscored the presence of alteration in severe insulin resistance and T2D conditions. This latter observation is in line with most studies reporting marked gut dysfunctions in late-stage disease models [8, 39–42].

Plasma citrulline levels are mainly determined by the enteric production rate from plasma amino acids precursors. The biosynthesis of citrulline per cell is constant, which led to the postulate that it reflects the absolute number of enterocytes. Indeed, contribution of the hepatic urea cycle is negligible on plasma citrulline levels and its excretion remain relatively stable in absence of kidney disease. Despite that none of the subjects were characterized with advanced kidney disease estimated with the CKD-EPI equation (GFR < 60 mL/min/1.73 m^2^), we still observed a correlation between the GFR and plasma citrulline levels. It suggests that even a physiologic reduction in kidney function tends to promote higher plasma citrulline levels. GFR is negatively associated with adiposity and metabolic complications, so that higher plasma citrulline levels may be expected in obese and diabetic subjects. In contrast to previous studies, all statistics have been adjusted for GFR to discriminate the independent contribution of small intestine mucosa of plasma citrulline levels. Indeed, kidney function may not impact the assessment of drastic change in small intestine mucosa mass (i.e., short-bowel syndrome, partial small intestine resection) as previously reported, but could counteract identification of subtle changes in small intestine mucosa mass such as those expected in obesity or diabetes.

Ramírez-Zamora et al. [43] highlighted an inverse correlation between plasma citrulline levels and glucose homeostasis in nondiabetic subjects. In contrast, positive associations between plasma citrulline, fasting glycemia and HbA1c in severely obese subjects were documented [4]. This study corroborates both observations, as citrulline levels were reduced in presence of glucose homeostasis alterations but elevated in diabetic subjects with abnormal fasting glucose and triglyceride levels. Nevertheless, men with T2D but normal TG and HbA1c values show lower citrulline levels than nondiabetic men. This observation may be related to the use of insulin sensitizing therapy which has been previously reported, in some case, as soon as the first utilization [44–46]. Without treatment, individuals with T2D were more likely to have higher citrulline levels than their nondiabetic counterpart [43]. In sum, T2D appears to be associated with increased small intestinal enterocyte mass especially in presence of elevated fasting glucose and triglyceride levels. Insulin sensitizing therapy appears to normalize small intestine enterocyte mass while this observation could be indirectly mediated by the availability of precursors to citrulline synthesis. In contrast, decreased insulin sensitivity in men without diabetes is apparently associated with reduced small intestine enteric mass.

Plasma levels of I-FABP are known to be a reliable biomarker of small intestine epithelial cell homeostasis in various enteropathies in adults and children [14, 21, 47]. Taking into consideration the very short half-life of this protein in circulation, the steady state circulating levels of I-FABP represents continuous release from senescent enterocytes. In our study, plasma I-FABP levels were elevated in men with T2D harboring uncontrolled glycemia and lipemia. These data are in line with Verdam et al. [4] observations in men with obesity and elevated glycated hemoglobin levels. These results put forward an increased cell death rate in presence of severe glucose homeostasis alterations. On the other hand, absence of association in men without T2D suggests that physiological variations in anthropometric measures, glucose homeostasis and metabolic profile have no impact on small intestine epithelial cell death. Correlation with FABP2 gene expression in the duodenum of these men without T2D further suggests that plasma I-FABP levels may be more related to enterocyte protein content than to the cell death rate itself.

The I-FABP-to-citrulline ratio was computed as an index of cellular turnover considering total enterocyte mass. Interestingly, the I-FABP-to-citrulline ratio significantly increased with impaired glucose homeostasis up to T2D states. This indicates that the enterocyte death rate is consistently more elevated than the small intestine mucosa mass in presence of metabolic complications. At equilibrium, this insinuates a higher cell renewal rate (i.e., increased cell death and differentiation rates) in presence of metabolic complications. This higher cell renewal rate is observed in both insulin-resistant and diabetic states while these conditions display distinct small intestine mucosa morphology.

Results of this study are strengthened by the use of a relatively large study samples of men covering a large spectrum of adiposity and glucose homeostasis status compared to previous studies. The present analyses are based on cross-sectional data and the difficulty to obtain intestinal samples from subjects without enteropathies limits the possibility of direct measurements. While it is established that 80–90% of the citrulline is derived from enterocytes [48], we cannot completely exclude that other secondary source of citrulline may explain these results. Finally, since we only had access to a well-characterized cohort of men, we cannot extrapolate our results to women small intestine morphologies. Nevertheless, no sex dimorphism was reported to date on I-FABP and citrulline levels as well as on small intestine mucosa morphology.

## Conclusion

In conclusion, our results support the hypothesis of a decreased functional enterocyte mass and a higher enterocyte death rate in presence of metabolic alterations. We also emphasized that small intestine epithelial cell homeostasis is especially altered in presence severe insulin resistance and T2D conditions. The marked changes in small intestine cellularity observed in obesity and diabetes are thus suggested to be part of gut dysfunctions, mainly at an advanced stage of the disease.

## Supplementary information

**Additional file 1: Table S1.** Physical and metabolic characteristics of samples of men with intestinal biopsies (n = 101).

## Data Availability

The datasets analysed during the current study are available from the corresponding author on reasonable request.
