# Supplementary material for: Plasma biomarkers of small intestine adaptations in obesity-related metabolic alterations
Source: Diabetol Metab Syndr. 2020 Apr 9;12:31. doi: 10.1186/s13098-020-00530-6 (PMC7144049; doi:10.1186/s13098-020-00530-6)
Supplement: Supplementary file 1 — Additional file 1: Table S1. Physical and metabolic characteristics of samples of men with intestinal biopsies (n = 101). [file 13098_2020_530_MOESM1_ESM.docx]

**Table S1: Physical and metabolic characteristics of samples of men with intestinal biopsies (n=101).**

| Variables | (Mean ±SD) |
| --- | --- |
| Anthropometrics |  |
| Age (years) | 38.7 ± 11.4 |
| BMI (kg/m^2^) | 31.5 ± 4.7 |
| Waist circumference (cm) | 108 ± 14 |
| Glucose homeostasis |  |
| Fasting glucose (mmol/L) | 5.3 ± 0.5 |
| HOMA-IR | 4.1 ± 1.7 |
| Insulin (pmol/L) | 119 ± 46.9 |
| Lipid profile |  |
| HDL cholesterol (mmol/L) | 1.1 ± 0.2 |
| LDL cholesterol (mmol/L) | 3.2 ± 0.9 |
| TG (mmol/L) | 2.1 ± 1.0 |
| hsCRP (mg/L) | 2.7 ± 3.7 |
